# Crystal growth of Dirac semimetal ZrSiS with high magnetoresistance and mobility

**DOI:** 10.1038/srep40603

**Published:** 2017-01-18

**Authors:** Raman Sankar, G. Peramaiyan, I. Panneer Muthuselvam, Christopher J. Butler, Klauss Dimitri, Madhab Neupane, G. Narsinga Rao, M.-T. Lin, F. C. Chou

**Affiliations:** 1 Institute of Physics, Academia Sinica, Taipei, 10617, Taiwan; 2 Center for Condensed Matter Sciences, National Taiwan University, Taipei 10617, Taiwan; 3 Department of Physics, National Taiwan University, Taipei 10617, Taiwan; 4 Department of Physics, University of Central Florida, Orlando, Florida 32816, USA; 5 National Synchrotron Radiation Research Center, Hsinchu 30076, Taiwan; 6 Taiwan Consortium of Emergent Crystalline Materials, Ministry of Science and Technology, Taipei 10622, Taiwan

## Abstract

High quality single crystal ZrSiS as a theoretically predicted Dirac semimetal has been grown successfully using a vapor phase transport method. The single crystals of tetragonal structure are easy to cleave into perfect square-shaped pieces due to the van der Waals bonding between the sulfur atoms of the quintuple layers. Physical property measurement results including resistivity, Hall coefficient (R_H_), and specific heat are reported. The transport and thermodynamic properties suggest a Fermi liquid behavior with two Fermi pockets at low temperatures. At T = 3 K and magnetic field of *H*ǁ*c* up to 9 Tesla, large magneto-resistance up to 8500% and 7200% for *I*ǁ_(*100*)_ and *Iǁ*_(*110*)_ were found. Shubnikov de Haas (SdH) oscillations were identified from the resistivity data, revealing the existence of two Fermi pockets at the Fermi level *via* the fast Fourier transform (FFT) analysis. The Hall coefficient (R_H_) showed hole-dominated carriers with a high mobility of 3.05 × 10^4^ cm^2^ *V*^*−1*^ *s*^*−1*^ at 3 *K*. ZrSiS has been confirmed to be a Dirac semimetal by the Dirac cone mapping near the **X**-point *via* angle resolved photoemission spectroscopy (ARPES) with a Dirac nodal line near the Fermi level identified using scanning tunneling spectroscopy (STS).

For the past few years, a variety of Dirac and Weyl semimetals including Cd_3_As_2,_ Na_3_Bi, TaAs, and NbAs, topological insulators including Bi_2_Se_3_, Bi_2_Te_3_ and LuPtBi, and samples of unusually high magnetoresistance including Cd_3_As_2_ and TaAs have been theoretically predicted and realized experimentally^1^,^2^,^3^,^4^,^5^,^6^,^7^,^8^,^9^,^10^. On the fundamental physics aspect, more interesting phenomena have been identified, such as the unsaturated large linear magnetoresistance (MR) with high mobility in high magnetic field, which have been proposed to originate from the linear dispersion at the band touching points with modulated electronic accumulation in the lowest Landau level^11^,^12^,^13^. In the search for materials with potential application, materials with extremely large and linear MR could be used for magnetic sensor and memory devices^14^. These materials hosting a low carrier density of compensated electrons and holes lay foundations in the exploration of new frontiers in the field of condensed matter physics^15^.

Recently, angle-resolved photoemission spectroscopy (ARPES) studies have identified the presence of various shapes of topological semimetal phase in ZrSiS, including a Dirac line node phase, a diamond-shaped Fermi surface at the Brillouin zone center (**Γ**) point, an ellipsoidal-shaped Fermi surface at the **M** point, and small electron-like pockets at the **X** point^15^,^16^. Recent magneto-transport studies revealed that ZrSiS exhibits an extremely large and anisotropic MR, as well as three dimensional and quasi-two dimensional Dirac Fermi surfaces with a topological phase transition^16^,^17^. The de Haas–van Alphen (dHvA) and Shubnikov de Haas (SdH) quantum oscillation studies revealed the essential properties of Dirac fermions, including bulk Dirac bands with non-trivial Berry phase, high mobility, and low effective mass^18^,^19^,^20^,^21^,^22^. Since the ZrSiS crystal possesses linear band dispersion within a wide energy range about 2 eV of the Dirac bands, which is much larger than other well-known Dirac semimetals, a comparative transport property study of ZrSiS would be very instructive.

In this work, we report the growth of large high quality single crystals of ZrSiS, and the characterization of their crystal structural, transport, and thermodynamic properties. An unsaturated large MR was observed at 3 *K* under the applied magnetic field up to 9 T. The observation of SdH quantum oscillation revealed the existence of two Fermi pockets. Angle-resolved photoemission spectroscopy (ARPES) revealed the multiple Fermi pockets with different dimensions of Fermi surface. Specific heat capacity study shows that ZrSiS has a higher Debye temperature than those of the typical Dirac and Weyl semimetals, such as Cd_3_As_2_^23^ and TaAs^24^.

## Results and Discussion

**Figure 1:**
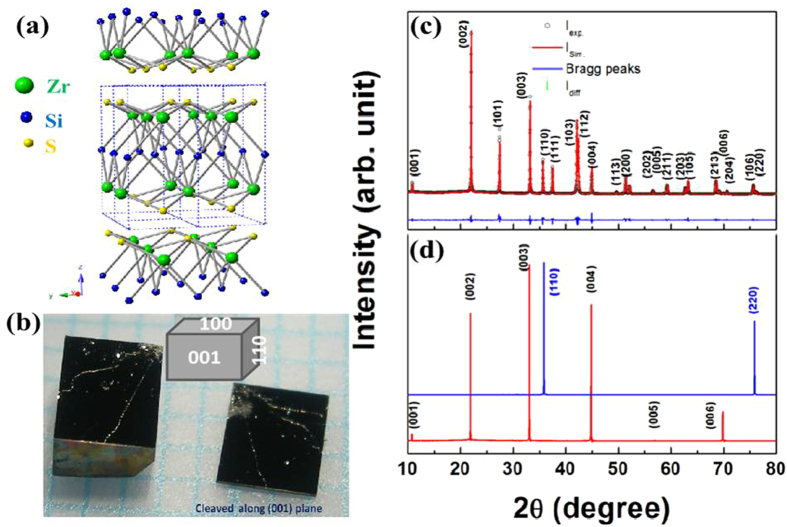
(**a**) Crystal structure of ZrSiS with the proposed cleavage plane between the S-layers. (**b**) Photograph image of typical ZrSiS single crystals with morphology of indexed crystallographic planes. (**c**) The Rietveld refinement of X-ray powder diffraction pattern of ZrSiS. (**d**) X-ray diffraction pattern of a ZrSiS single crystal in two preferred orientations of (001) and (110).

The crystal structure of ZrSiS can be described as a typical layered compound of quintuple layers of S-Zr-Si-Zr-S, as shown in Fig. 1(a). Each quintuple layer is centered around the Si which bonds four Zr atoms in tetrahedral coordination, and the S atoms are on the two sides of each quintuple layer, weakly bonding with the neighboring layers *via* a van der Waals type interaction. Based on the molecular orbital analysis, the electronic structures of the Zr ([Kr]4d^2^5s^2^) of 8-fold coordination and the S ([Ne]3s^2^3p^4^) of 4-fold coordination imply imbalanced numbers between the valence electron and coordination number, which strongly suggests the existence of Zr*-d* and S-*p* orbital hybridization of intermediate metallicity, in agreement with the calculated band structure for a Dirac semimetal^16^,^22^,^25^,^26^.

Figure 1(b) shows a typical image of the as-grown single crystals of ZrSiS. Based on the X-ray diffraction patterns, the crystal surfaces can be identified as the low indexing planes of (100), (110) and (001) and the inset shows the morphology of the as-grown ZrSiS single crystal. Figure 1(c) shows the Rietveld refinement of the X-ray powder diffraction results (Bruker D8) using Cu*-K*_*α*_ radiation for the pulverized single crystal sample. All of the diffraction peaks can be indexed with the tetragonal system of space group *P4/nmm*. The refined lattice parameters are *a* = *b* = 3.5440 *Å, c* = 8.0550 *Å*, and volume (V) = 101.17*Å*^3^, which are in good agreement with those reported in the literature^25^. Figure 1(d) shows the typical X-ray diffraction patterns for the single crystal sample with two planes of preferred (00 l) and (110) orientations.

**Figure 2:**
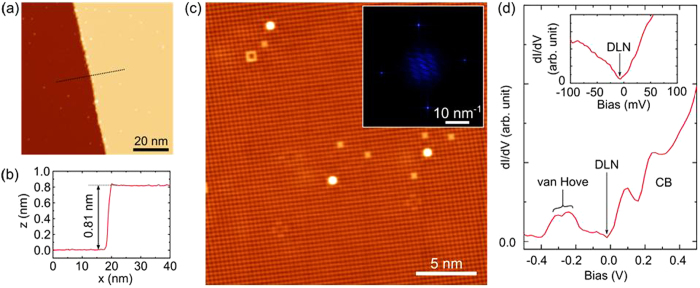
STM and STS measurements on vacuum-cleaved ZrSiS. (**a**) Large scale STM topography including a step-edge, with the topographic line profile displayed in (**b**), showing a step height of 0.81 nm, consistent with the ZrSiS c-axis lattice parameter. (**c**) High-resolution STM topography showing the square sulfur surface lattice of ZrSiS, and its 2-D FFT (inset). (Large-scale and zoom-in topography taken using set-point V = 0.2 V, I = 0.3 nA). (**d**) Tunneling spectroscopy curve, spatially averaged over the same area shown in (**c**) (set-point V = 0.2 V, I = 0.3 nA, lock-in V_mod_ = 10 mV). The pair of peaks between −0.2 and −0.4 eV is attributed to van Hove singularities corresponding to the stationary points of the surface state. A higher energy-resolution spectrum around E_F_ (inset) has a ‘V’-shaped minimum at −8 mV, which may correspond to the Dirac line nodes (DLN) along both the surface projected 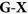 and 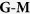 directions (set-point V = 50 mV, I = 0.5 nA, lock-in V_mod_ = 1 mV). The conduction band (CB) onset occurs above E_F_.

STM measurements were performed on a ZrSiS platelet which was cleaved at room-temperature in a vacuum atmosphere better than 5 × 10^−11^ mbar, then quickly transferred into an Omicron LT-STM for measurement at a temperature of 4.5 K. STM measurements were performed using an electrochemically etched tungsten tip. d*I/*d*V(V*) spectroscopy curves were acquired using the lock-in technique with a bias modulation, as stated in the relevant figure caption. The (001)-oriented cleavage plane is expected to be between the two adjacent S atomic layers. Figure 2(a) shows large-scale STM topography of the vacuum-cleaved surface revealing a step-edge of height of 0.81 nm, as shown in Fig. 2(b), consistent with the perpendicular spacing between cleavage planes, *i.e.* the c-axis lattice parameter. Figure 2(c) reveals the square S surface lattice with no discernible reconstruction. A number of round bumps can be seen, most likely adatoms resting at hollow sites on top of the S surface lattice, which may be debris resulting from the cleavage process. Numerous surface defects are also seen, representing possible H interstitials between S atoms of the surface lattice, and probable substitutions occupying various lattice sites. Figure 2(d) shows tunneling spectroscopy with a prominent double-peaked feature in the range between −0.2 and −0.4 eV. This feature may correspond to a pair of van Hove peaks at the stationary points of the ‘*M*’-shaped feature of the surface state around the Brillouin zone’s **X** point, as revealed in the ARPES results reported by Schoop *et al*.^16^. The d*I/*d*V* minimum slightly below *E*_*F*_ may correspond to the LDOS minimum associated with the Dirac crossings along both the surface projected 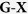 and 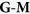 directions. The onset of the bulk conduction bands is seen above *E*_*F*_.

**Figure 3:**
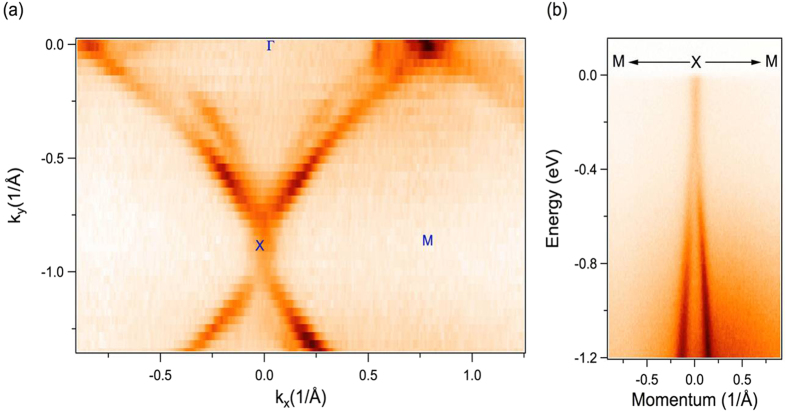
ARPES characterization of ZrSiS. (**a**) ARPES measured Fermi surface map, with high-symmetry points marked on the plot. Various Fermi pockets are observed as described in the text. (**b**) ARPES dispersion map along M-X-M momentum space direction. These spectra were measured at photon energy of 70 eV at T = 18 K at the Swiss light source, PSI.

Spectroscopic characterization of the single crystal ZrSiS was carried out using angle-resolved photoemission spectroscopy (ARPES). Systematic high-resolution ARPES measurements for the low-energy electronic structure investigation were performed at the SIS-HRPES end station at the SLS, PSI, Switzerland, which is equipped with a high-efficiency R4000 electron analyzer. The energy and momentum resolutions were better than 40 *meV* and 1% of the Brillouin Zone (BZ), respectively. The samples were cleaved *in-situ* and measured at 18 *K* in a vacuum better than 10^**−**10^ torr. The crystals were found to be very stable, exhibiting no degradation for the typical measurement period of 20 hours. Figure 3(a) shows the Fermi surface map measured with incident photon energy of 70 *eV* at T = 18 *K*. Our wider BZ mapping reveals multiple Fermi surface pockets on the surface of ZrSiS crystals. The observed pockets include a diamond-shaped Fermi surface, an elliptical-shaped Fermi surface, and a small electron pocket, encircling the zone center (**Γ**) point, the **M** point, and the **X** point of the *BZ*, respectively (see Fig. 3(a)). Furthermore, a Dirac-like dispersive state mostly originating from the surface is observed at the **X** point, as shown in Fig. 3(b). We note that these results are consistent with the recently published observations^16^,^17^. The observed sharp ARPES spectra provide a strong evidence to indicate the high quality of single crystals.

**Figure 4:**
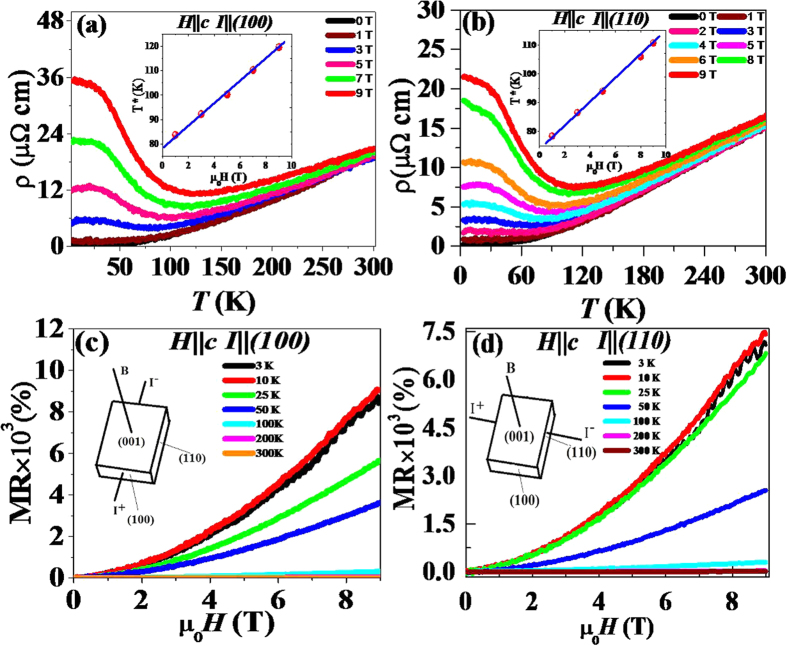
(**a**,**b**) Temperature dependent resistivity in selected magnetic fields H//c with the current *I*‖(*100*) and *I*‖(*110*), respectively. Insets of (**a**,**b**) show the linear dependence of the crossover temperature T* on magnetic field. (**c**,**d**) Magnetoresistance at different temperatures with the current *I*‖(*100*) and *I*‖(*110*), respectively. SdH oscillations are observed at high field. The insets show the measurement geometry.

The resistivity and magnetoresistance measurement results of single crystal ZrSiS, in magnetic fields of *μ*_*0*_*H (H*‖*c*) up to 9 Tesla for current flow along the (100) and (110) directions are shown in Fig. 4(a,b). Metallic behavior is observed for both configurations in the absence of magnetic field, down to a temperature of 3 *K*. The room temperature resistivities are found to be 15 and 19.4 *μΩ-cm*, and fall to 0.18 and 0.38 *μΩ-cm* at 3 *K*, and their corresponding residual resistivity ratios (RRR = ρ_300K_/ρ_3K_) are 83 and 51 for *I*‖(100) and *I*‖(110), respectively, which attests to the high quality of the grown single crystal sample. The resistivity of ZrSiS at low temperature is lower than the recently reported semimetals WTe_2_^27^ and NbP^13^. When a field is applied, the resistivity of the sample follows the metallic behavior until it approaches certain temperature called the “crossover” temperature T* (defined by the resistivity minimum) for metallic to semiconducting systems (insets of Fig. 4(a,b)), below which the resistivity begins to increase dramatically, which is similar to the metal-semiconductor transition induced by the magnetic field, as also observed in WTe_2_^27^, PdCoO_2_^28^, and NbSb_2_^29^.

Figure 4(c,d) shows the magnetoresistance (MR) measured in the *I*‖_(100)_ and *I*‖_(110)_ directions as a function of magnetic field up to 9 *T* at different temperatures. The MR percentage, calculated from [ρ(H) − ρ(0)]/ρ(0)] × 100%, reaches 8500*%* and 7200% at 3 *K* without any signature of saturation in a field of 9 T for *I*‖_(100)_ and *I*‖_(110)_, respectively. The observed MR at low-temperature is nearly one order lower than those reported for TaAs and WTe_2_^3^,^27^. The large and unsaturated MR of ZrSiS may be interpreted as a quantum effect due to the linear energy dispersion at the band touching point^13^. Upon raising the temperature, the MR of ZrSiS remains almost unchanged at low-temperature and then begins to decrease drastically at higher temperatures for both the *I*‖_(100)_ and *I*‖_(110)_ directions. Scaling of magnetoresistance (MR) data for different temperatures under Kohler’s rule 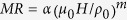 shows that these curves do not fall into a single curve, indicating the violation of Kohler’s rule as shown in Fig. S8(a) and (b). It means that the ZrSiS crystal has more than one type of charge carriers^18^,^30^.

**Figure 5:**
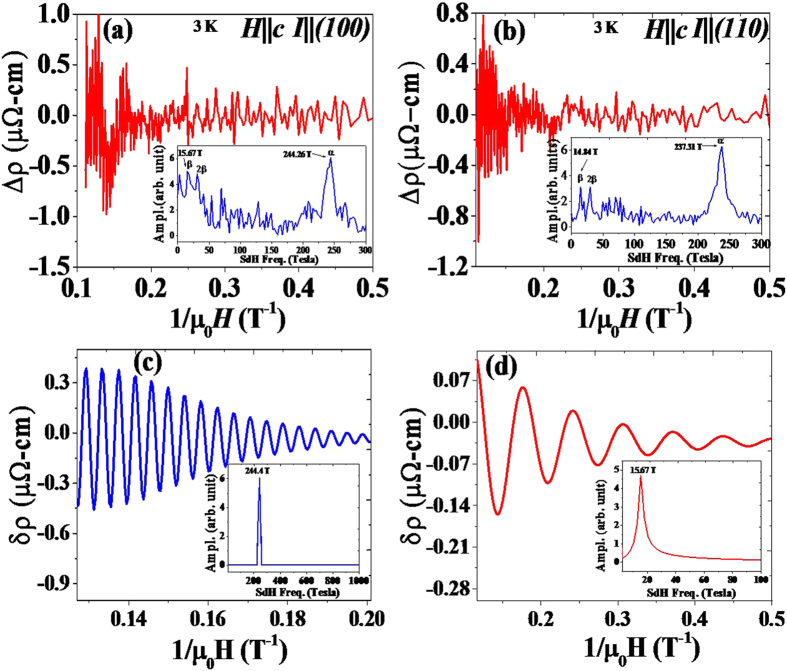
The resistivities at 3 K after the polynomial background subtraction, for (**a**) I‖(100) and (**b**) I‖(110). The corresponding FFT spectra are shown in the insets. FFT filtering separated oscillation patterns for I‖(100), δρ, for the frequencies of (**c**) 244.26 T and (**d**) 15.67 T. The corresponding FFT spectra are shown in the inset.

Strong Shubnikov de Haas (SdH) oscillations are observed in the *I*‖_(100)_ and *I*‖_(110)_ directions at 3 K, as shown in Fig. 4(c) and (d), providing added evidences for the coexistence of multiple Fermi surfaces. In order to analyze the SdH oscillations, a smoothed background is subtracted from the raw *ρ(H, T*) data to obtain the oscillatory component (Fig. 5(a,b). A fast Fourier transform (FFT) analysis is performed to extract the frequency spectrum of the SdH oscillations, as shown in the inset of Fig. 5(a,b). The obtained FFT spectra show two distinct peaks at 14.84 T and 237.31 T for *I*‖_(110)_ and 15.67 T and 244.26 T for *I*‖_(100)_, corresponding to two Fermi pockets and the obtained results are consistent with the previous studies^18^,^19^. We used FFT filtering to separate the oscillation patterns (denoted as δρ) for the observed frequencies of 244.26 T and 15.67 T for *I*‖_(100)_ direction as shown in Fig. 5(c,d), and the insets show the obtained FFT frequencies verified from the extracted δρ patterns. Using the Onsager relation, 
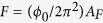
, where the *A*_*F*_is the Fermi surface area cross section perpendicular to the field, F is the frequency of oscillation, and *φ*_0_ is the magnetic flux quantum, the Fermi surface cross sections are calculated to be 1.41 × 10^−3^* Å*^−2^ and 22.63 × 10^−3^*Å*^*−2*^ for 14.84 T and 237.31 T, 1.49 × 10^−3^ *Å*^*−2*^ and 23.30 × 10^−3^ *Å*^*−3*^ for 15.67 T and 244.26 T, respectively, which are close to the values reported in the literature^18^,^20^,^21^.

**Figure 6:**
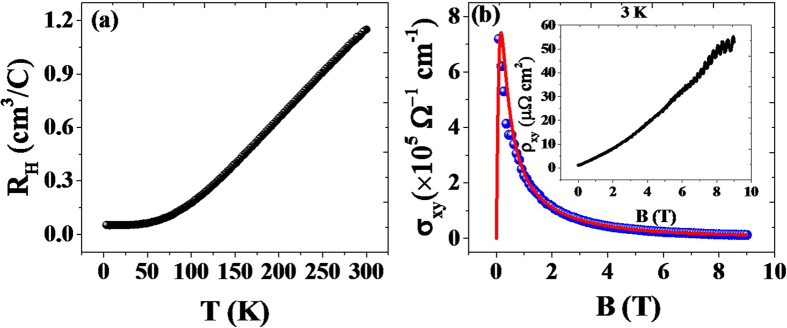
(**a**) Hall coefficient (*R*_*H*_) as a function of temperature, (**b**) Magnetic field dependence of Hall conductivity at 3 K. The solid line is the two-band model fitting calculation using Eq. 1, which yields the carrier concentration and mobility for the electrons and holes (n_e_, n_h_, μ_e_ and μ_h_), and inset shows the field dependence of Hall resistivity, ρ_xy_.

Hall coefficient (*R*_*H*_) measurements were performed to investigate the carrier mobility and concentration. The temperature dependence of R_H_ is shown in Fig. 6(a) for a ZrSiS single crystal under an applied magnetic field of 5000 *Oe*. At low temperature *R*_*H*_ and field dependent Hall resistivity data at 3 K (inset of Fig. 6(b)) apparently deviates from the linearity indicating the two types of carriers, and the positive value throughout the measured temperature range implies that the conduction mechanism is hole-dominated and decreases with decreasing temperature from 300 *K* to 60 *K* until it saturates to a constant.

Using the two band model^31^, the carrier densities and mobilities can be calculated by fitting the field dependent Hall conductivity (σ_xy_) data,


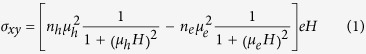


where 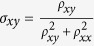, and n_e_ (n_h_) and μ_e_ (μ_h_) are the carrier concentrations and mobilities of electron and holes, respectively. The resistivity (ρ_xx_) of *I*‖_(100)_ direction was used for the Hall conductivity calculation. From the fitting results at 3 K as shown in Fig. 6(b), n_h_ = 1.55 × 10^23^ *cm*^*−3*^, n_e_ = 1.12 × 10^23^ *cm*^*−3*^, μ_h_ = 5.9 × 10^4^ *cm*^2^ *V*^*−1*^ *s*^*−1*^ and μ_e_ = 0.20 × 10^4^* cm*^*2 *^*V*^*−1*^ *s*^*−1*^ were obtained. The average mobility, μ = 3.05 × 10^4^ *cm*^*2*^ *V*^*−1*^ *s*^*−1*^ is very close to the already reported data (1.79 × 10^4^ *cm*^*2*^ *V*^*−1*^ *s*^*−1*^)^19^ of ZrSiS crystal. The carrier concentration of ZrSiS crystal is four orders of magnitude larger than that of the topological Weyl semimetal NbP. The estimated mobility at 3 *K* is two orders of magnitude lower than the compounds in the same class of semimetals, including Cd_3_As_2_ (9 × 10^6^ cm^2^/V·s, at 5 K)^7^, NbP(5 × 10^6^ *cm*^*2*^*/V*·*s*, at 1.85 *K*)^12^, but close to that of TaAs (5 × 10^5^ *cm*^*2*^*/V*·*s*, at 2 K)^3^.

**Figure 7:**
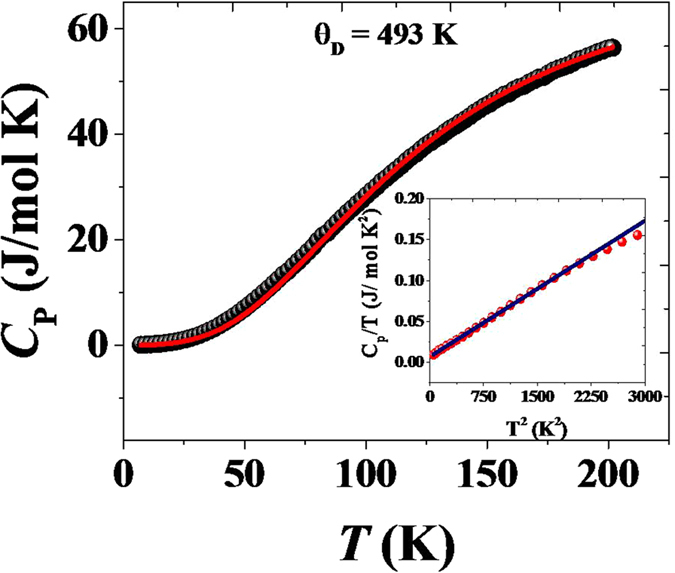
Specific heat of ZrSiS single crystal. The solid line is the fit to the Debye model using the Eq. 2. Inset shows the *C*_*P*_*/T* data fitted with the expression of 
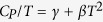
.

The electronic properties were explored further with the specific heat measurement. Figure 7 shows the specific heat (*C*_*P*_) of a ZrSiS single crystal from 0 to 200 K. It can be seen that *C*_*P*_starts to saturate at 57 J mol^−1^ K^−1^ above ~200 *K*, which is close to the Dulong-Petit value, and an exponential suppression of specific heat at low temperatures is observed. The solid line is the fit to the Debye model^24^,


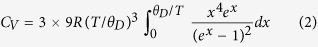


where *θ*_*D*_ is the Debye temperature and R the ideal gas constant. By fitting the above equation, the θ_D_ is obtained to be ~493 K, which is the largest among those observed in the typical Dirac and Weyl semimetals, including Cd_3_As_2_ (200 *K*), TaAs (352 *K*) and pyrochlore oxides (400 *K*)^23^,^24^,^32^,^33^. The inset of Fig. 7 shows the *C*_*P*_*/T* data fitting below 55 *K* using the expression of 
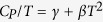
, where the T linear term in *C*_*p*_ represents the electronic contribution and the higher order term represents the lattice contribution. The derived Sommerfeld coefficient *γ* is estimated to be 6.84 *mJ mol*^*−1*^ *K*^*−2*^, which is higher than several other typical semimetals including NbAs (0.09(1) mJ mol^−1^ K^−2^)^34^, Y_3_Ir_4_Ge_13_ (4.3 mJ mol^−1^ K^−2^)^35^, and YPtBi (4 mJ mol^−1^ K^−2^)^36^. The phononic contribution is estimated to be *β* = 5.54 × 10^−5^* J mol*^*−1*^ *K*^*−3*^. Since the Sommerfeld coefficient (*γ*) is proportional to the electronic density of states near the Fermi level (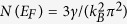, N(E_F_) is calculated to be 2.90 eV^−1^ fu^−1^.

## Conclusion

In summary, we have grown high quality single crystals of Dirac semimetal ZrSiS and performed a systematic physical and electronic property characterization to compare with some typical semimetals in the same class. Tunneling spectroscopy acquired at the cleaved (001) surface, visualized using STM, is consistent with previous ARPES observations of a distinct surface state and a Dirac line node near the Fermi level. Our ARPES measurements revealed the presence of multiple Fermi pockets of three different shaped Fermi surface with dispersion map along the **M-X-M** momentum space direction to substantiate the quality of the grown crystal sample. The large unsaturated MR up to 9 Tesla is found at 3 *K* and compared with those reported semimetals of the same class. ZrSiS exhibits strong Shubnikov de Haas (SdH) oscillations observed in magneto-resistance measurements. The positive value of the Hall coefficient observed between 3 and 300 *K* suggests *p* type carriers dominating ZrSiS, and the mobility is found to be 3.05 × 10^4^ cm^2^ V^−1^ s^−1^ comparable with that of TaAs.

## Experimental Section

### Sample Preparation

Single crystals of ZrSiS, as shown in Fig. 1(b), were grown in using a two-step chemical vapor transport process using I_2_ as the transport agent. A silica ampoule with length of 20–30 cm and ID/OD of 1.8/2.0 mm was used for the synthesis and growth. In the first step for direct solid state reaction, a stoichiometric amount of 5 N purity precursors of Zr:Si:S = 1:1:1 molar ratio was sealed in an evacuated quartz ampoule. The vacuum-sealed quartz ampoule containing ternary mixtures was treated at 1100 *°C* for one week and then furnace-cooled to room temperature. The polycrystalline ZrSiS sample was confirmed to be single phase. In the second step, the polycrystalline powder of ZrSiS was mixed with I_2_ in weight ratio of 100:1 and vacuum-sealed in a two-zone tube furnace having a thermal gradient of about 1100–950 *°C* within ~30 cm. After a period of 10 days, single crystals of ZrSiS with size up to 4.0 × 2.9 × 3.0 mm^3^ were obtained. The ZrSiS single crystals have shiny cleaved surface with a morphology of well-defined crystal planes indexed as (100), (110), and (001), as shown in the inset of Fig. 1(b).

## Additional Information

**How to cite this article**: Sankar, R. *et al*. Crystal growth of Dirac semimetal ZrSiS with high magnetoresistance and mobility. *Sci. Rep.* **7**, 40603; doi: 10.1038/srep40603 (2017).

**Publisher's note:** Springer Nature remains neutral with regard to jurisdictional claims in published maps and institutional affiliations.

## Supplementary Material

Supplementary Information
