# Supplementary material for: Crystal growth of Dirac semimetal ZrSiS with high magnetoresistance and mobility
Source: Sci Rep. 2017 Jan 18;7:40603. doi: 10.1038/srep40603 (PMC5241817; doi:10.1038/srep40603)
Supplement: Supplementary Information [file srep40603-s1.doc]

**Supplementary**

**Crystal growth of Dirac semimetal ZrSiS with high magnetoresistance and mobility**

Raman Sankar^1,2^,*, Peramaiyan Ganesan^1^, I. Panneer Muthuselvam,^1,2^ Christopher J. Butler,^3^ Klauss Dimitri^4^, Madhab Neupane^4^, G. Narsinga Rao^2^, M.-T. Lin^3^, and F. C. Chou^,2,5,6*^


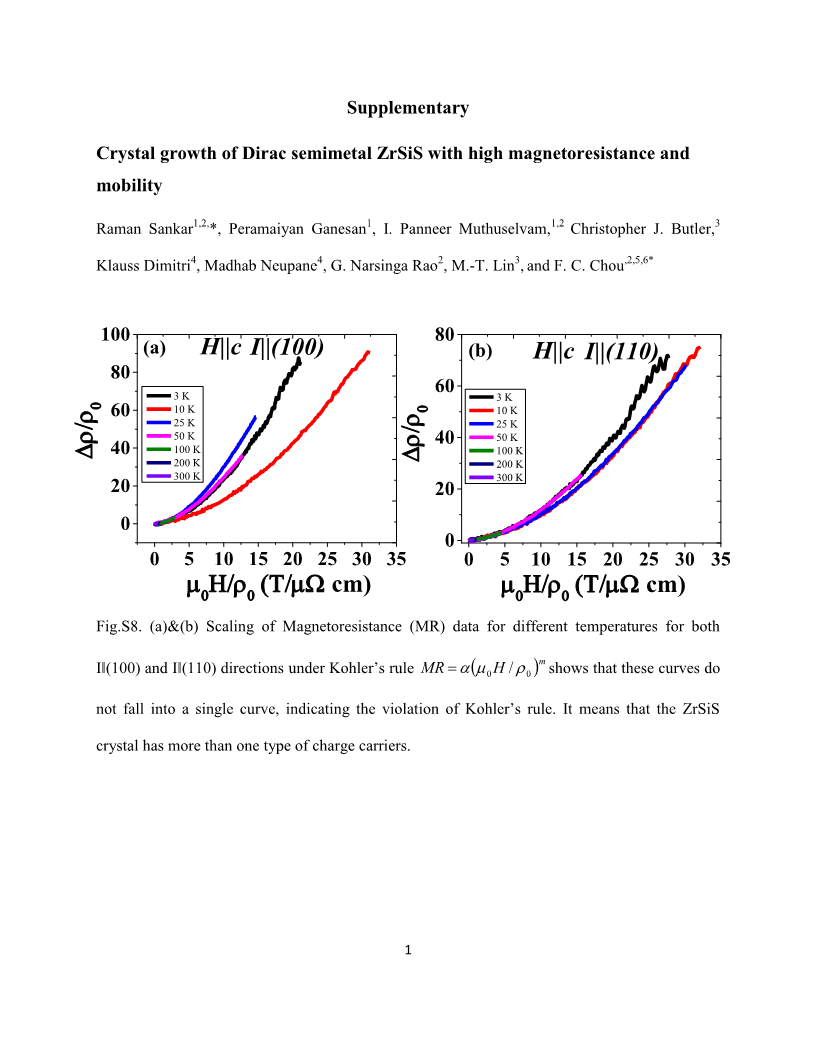


Fig.S8. (a)&(b) Scaling of Magnetoresistance (MR) data for different temperatures for both I‖(100) and I‖(110) directions under Kohler’s rule $MR=\alpha\left(\mu_{0}H/\rho_{0}\right)^{m}$shows that these curves do not fall into a single curve, indicating the violation of Kohler’s rule. It means that the ZrSiS crystal has more than one type of charge carriers.
